# Associations between air pollution and biomarkers of oxidative stress and lung damage in a large population-based sample of non-smoking adults in northern France

**DOI:** 10.1007/s10653-025-02472-2

**Published:** 2025-04-12

**Authors:** Raphaël Bentegeac, Djamal Achour, Céline Grare, Manon Muntaner, Victoria Gauthier, Philippe Amouyel, Regis Matran, Farid Zerimech, Jean-Marc Lo Guidice, Luc Dauchet

**Affiliations:** 1 U1167 - RID-AGE, INSERM, Lille, France; 2 Institut Pasteur de Lille, Lille, France; 3 Lille University Hospital Center, Lille, France; 4 Lille University, Lille, France; 5 ULR 4483 – IMPECS, Lille University, Lille, France

**Keywords:** Air pollution, Biomonitoring, Health studies, Population-based study, Transformation products/metabolites

## Abstract

Air pollution is an environmental risk factor associated with lung and cardiovascular disease that may be mediated by physiological pathways such as oxidative stress. Previous studies have identified associations between air pollution and biomarkers of oxidative stress (8-OHdG, 4-HNE, and fluorescent oxidation products (FOPs)), as well as lung health marker CC16, in younger and asthmatic populations. The objective of this study of a large population-based sample of non-smoking adults was to explore the relationship between long-term and short-term atmospheric pollution exposures and plasma or urine levels of these biomarkers. Our study was a post-hoc analysis of the cross-sectional ELISABET study from 2011 to 2013. We included non-smoking inhabitants of Lille, France from the ELISABET study. We assessed mean pluri-annual residential and short-term exposures to atmospheric pollution components (PM_10_, NO_2_, and O_3_) and collected several biomarkers (CC16, 8-OHdG, 4-HNE, and fluorescent oxidation products (FOPs)). We searched for associations between pollutants and biomarkers using log-linear robust multivariate regressions. Our work did not show any association between short- or long-term exposure to air pollution components and CC16, 8-OHdG, 4-HNE or FOP in a large (980 subjects) sample of Lille’s general population, despite having sufficient statistical power to replicate previous findings of associations between air pollution and these biomarkers found in younger or asthmatic populations.

**Supplementary Information:**

The online version contains supplementary material available at 10.1007/s10653-025-02472-2.

## Introduction

Air pollution is acknowledged to be a major public health problem because it leads to an increase in morbidity and mortality rates (Murray et al., 2020). The effects of air pollution on the cardiovascular (Chen & Hoek, 2020) and respiratory (Andersen et al., 2011) systems are particularly harmful.

The association between air pollution and disease is underpinned by a variety of pathophysiological pathways. We previously analyzed the association between air pollution and inflammation markers among participants in the ELISABET cohort study (Darras-Hostens et al., 2022). Chronic, low-grade, systemic oxidative stress and direct respiratory cell damage (Mu et al., 2021) are also thought to be involved.

With regard to oxidative stress, the air pollutants ozone (O_3_) and nitrogen dioxide (NO_2_) are strong oxidants (Rumble, 2021). Some of the chemical components of particulate matter (PM) also have oxidative properties.

Club cell secretory protein (CC16) is produced primarily by the non-ciliated bronchial epithelial cells. CC16 is thought to promote tissue repair, and reduced levels of this protein have been linked to asthma (Broeckaert et al., 2000; Laing et al., 2000). The presence of CC16 is correlated with good respiratory health, and smokers have low levels of CC16 (Hermans & Bernard, 1996).

The nucleoside 8-hydroxydeoxyguanosine (8-OHdG) is produced by oxidation of deoxyguanosine in cells subjected to oxidative stress, and blood and urine levels of 8-OHdG serve as oxidative stress markers (Valavanidis et al., 2009). Similarly, 4-hydroxynonenal (4-HNE) is a fatty acid peroxidation product, and the blood levels is also an oxidative stress marker (Awasthi et al., 2003; Raza & John, 2006). In vitro, 4-hydroxynonenal is associated with elevated apoptosis. In humans, 4-hydroxynonenal is associated with several diseases, such as Alzheimer’s disease, type 2 diabetes, chronic inflammation, and cancer (Pillon et al., 2012; Zarkovic, 2003). Lastly, fluorescent oxidation products (FOPs) reflect overall oxidative damage to cellular lipids, proteins, carbohydrates, and DNA (Wu et al., 2007).

Concerning CC16, associations with air pollution have been found in children and adolescents (Beamer et al., 2019; Provost et al., 2014). No associations were found in studies of adult populations, although the sample sizes were small: respectively 34 and 218 subjects (Zhang et al., 2022; Zuurbier et al., 2011). Likewise, 8-OHdG levels have been linked to air pollution in children (Svecova et al., 2009) and young adults (Lu et al., 2007) but not in a Chinese study of older adults (Mu et al., 2021). Although the putative link between air pollution and 4-HNE levels has been evaluated in a murine model (Cacciottolo et al., 2020; Cheng et al., 2016), we are not aware of any such studies in humans. FOP levels have been linked to air pollution in a population of people with asthma (Havet et al., 2019). However, a significant association was not observed in a small sample of healthy participants (Hart et al., 2012). Hence, previous studies in adults on associations between levels of air pollutant and these biomarkers in adults were based on small sample sizes.

The objective of the present study was to explore the associations between short term and long term residential atmospheric pollutant exposure (PM_10_, NO_2_, O_3_), and blood marker of systemic oxidative stress (4-HNE, FOP and 8-OHdG), and pulmonary damage biomarkers (CC16) in a large subsample of non-smokers from the ELISABET study population in the Lille urban area in France.

## Methods

The study population was derived from the *Enquête Littoral Souffle Air Biologie Environnement* (ELISABET) cross-sectional study in which recruitment was carried out between January 2011 and November 2013 in the Lille and Dunkirk areas of northern France. Study participants were aged between 40 and 65 and had lived in one of the two selected urban areas for at least 5 years. The participants were recruited from a random sample drawn from voter lists, stratified by age, urban area, and sex. Individual participant data were collected through a paper-based survey, which contained questions on lifestyle, diet, personal and family medical histories, age, and place of residence. As described previously (Darras-Hostens et al., 2022) in compliance with the French legislation on biomedical research, the study protocol was approved by the local investigational review board (CPP Nord Ouest IV, Lille, France; reference: 2010-A00065-34; ClinicalTrials.gov identifier: NCT02490553). All the participants provided their written, informed consent to participation in the study.

Surveys were conducted by nurses in the participant’s home or during consultations in a health facility. A blood sample was collected at the same time.

We excluded all smoking participants, in order to avoid bias due to the inflammation caused by smoking. We also excluded all individuals for whom data on pollution exposure were missing. As funding was only sufficient for approximately 1000 blood samples, we decided to evaluate participants from the Lille area because the standard deviation of the residential air PM_10_ level was higher in Lille than in Dunkirk (1.936 μg/m^3^ vs. 0.999 μg/m^3^, respectively), optimizing statistical power within our sample size constraints (Darras-Hostens et al., 2022).

The Lille urban area is dense; it encompasses the cities of Lille, Roubaix and Tourcoing and a few surrounding non-urban areas. The area is crossed by five highways, and the mean estimated daily flow of motorized vehicles was between 36,000 and 176,000 at the time of the study (“Cartes de Trafics Annuels,” n.d.). This area meets the “Improving Knowledge and Communication for Decision Making on Air Pollution and Health in Europe” (APHEKOM) criteria (Pascal, n.d.) and notably the criterion on homogeneous pollution exposure levels in each selected area.

Concerning the collecting and analysis of the blood and urine samples, all samples were added to ELISABET’s biobank at the time of inclusion (2011 to 2013). CC16, 8-OHdG, 4-HNE and FOPs were measured on these samples in 2020.

For the plasmastic CC16 assay, we used the Human Uteroglobin DuoSet ELISA Kit (DY4218, R&D Systems, Minneapolis, MN) and the DuoSet ELISA Ancillary Reagent Kit (DY008, R&D Systems) according to the manufacturer's instructions. The plate was read on a Spark® Multimode Microplate Reader (Tecan, Männedorf, Switzerland), and the data was analyzed with GraphPad Prism software (version 8.0.2, GraphPad Software LLC, San Diego, CA, USA). The CC16 level was expressed in pg/mL.

For the urine 8-OHdG assay, we used the OxiSelect Oxidative DNA Damage ELISA Kit (STA-320, Cell Biolabs, San Diego, CA) according to the manufacturer's instructions. The plate was read on a Spark® Multimode Microplate Reader (Tecan), and the data were analyzed with GraphPad Prism software. The 8-OHdG level was expressed in ng/mL urine.

For the plasmastic 4-HNE assay, we used MesoScale Discovery Electrochemiluminescence technology with Abcam 4HNE protein and a secondary antibody. Briefly, plates (L15XA-3, MesoScale Discovery, Rockville, MD) were coated one night at 4 °C with 4-HNE-BSA (ab194193, Abcam, Cambridge, United Kingdom). After washing three times with PBS-T, the plate was blocked one hour at RT with 3% of Blocker A (R93BA-4, MesoScale Discovery). Next, after 3 washes, samples or standard (4-HNE BSA) and the Anti-4 Hydroxynonenal Rabbit polyclonal Antibody (ab46545, Abcam) were added for one hour at RT. After incubation and three washes, Anti Rabbit Antibody Sulfo-TAG Labeled R32AB-5 was added. Finally, the reading buffer (R92TG-1, MesoScale Discovery) is added and the plate is read immediately on the MESO QuickPlex SQ 120MM Instrument and analyzed using Discovery Workbench 4.0 software (both from MesoScale Discovery). The 4-HNE level was expressed in pg/mL.

For quantification of the FOPs, the latter were extracted from plasma with an ethanol/ether mixture (3:1, v/v). Briefly, in borosilicate glass tubes, 0.2 mL of plasma was added to 1 mL of Mix. The tube is vortexed vigorously for 30 s then centrifuged for 10 min at 3000 rpm at 4 °C. The standard is prepared by diluting quinine in sulfuric acid at the following concentrations: 1, 0.5, 0.25, 0.1, 0.05 and 0.025 µg/mL. 200 µL of duplicate standard or 200 µL of sample supernatant is deposited on the plate and the fluorescence was read at three pairs of excitation/emission wavelengths: 320/430 nm (oxidation of linoleate with DNA in presence of metals), 360/430 nm (lipid oxidation products that have reacted with proteins, DNA and carbohydrate), and 400/465 nm (Malondialdehyde reacting with proteins and phospholipid) on the Spark® Multimode Microplate Reader (Tecan). In addition, until reading plate, all steps are carried out on ice, and the plate is read every third sample, as the supernatant is highly volatile. The FOP level was expressed as the fluorescence intensity per mL of plasma.

The methods for estimating exposure to air pollutants in the ELISABET project have been described in detail elsewhere (Darras-Hostens et al., 2022; Dauchet et al., 2018; Quach et al., 2015; Riant et al., 2018). Briefly, we estimated both residential and short-term exposures to pollutants.

Short-term exposure was defined as the mean level of the pollutant studied in the Lille urban area on the day of the biomarker sampling and the day before. This variable corresponds to short-term and supposedly reversible impact of pollution peaks.

Short-term exposure was estimated through daily measurements of the PM_10_ and NO_2_ concentrations and hourly measurements of the ozone (O_3_) concentration from air pollution monitoring stations managed by the ATMO Hauts-de-France association (Lille, France). The data from these stations met the APHEKOM homogeneity criteria (Pascal, n.d.). Essentially, this implies that most of the population should reside and work within the study area. The area must not be significantly affected by a large, stationary source of pollution that could create substantial variability in pollution levels. Additionally, the urban study area should remain continuous, without being interrupted by extensive non-urban spaces, and it should not exhibit major topographical variations that could influence the dispersion and concentration of pollutants. To facilitate the visualization and analysis of the characteristics of the two urban areas, we employed a web-based tool (“Géoportail,” n.d.) to map roads, urbanization levels, topography, and the distribution of atmospheric pollution monitoring stations.

Daily pollution levels for each urban area were defined as the average of the day’s concentrations for NO_2_ and PM_10_, and the average of the 8 h of the day with the highest O_3_ concentrations.

For residential exposure to air pollutants, the annual averages of PM_10_ and NO_2_ concentrations measured between 2010 and 2013 in Lille were retrieved from the estimates produced by ATMO Hauts-de-France using an air dispersion modeling system (ADMS version 3.4). The model did not provide an estimation of residential exposure to ozone. Furthermore, the model included meteorological, topographical and land use data, pollutant emissions from natural and man-made sources, and the measurements from several monitoring stations. The model allowed the production of annual average concentration maps for the studied urban areas; we used a spatial resolution with cells of 25 × 25 m. All participants had their home address placed in this grid. We considered the annual exposure to be the mean of the value of the residence cell and that of the four adjacent cells, weighted by the inverse square of the distance between the center of the cell and the exact location of the residence.

Residential exposure each participant was then calculated as the average level of pollution over the year of their inclusion and the years before. This variable corresponds to the impact of chronic exposure to air pollutants.

Of the individual variables available through the ELISABET survey questionnaire, we analyzed the age (in years) and the body mass index (in kg/m^2^). Some studies have shown that weather conditions are associated with both air pollution levels (Lin et al., 2021) and air-pollution-related diseases (Gauer & Meyers, 2019; Hansel et al., 2016). Accordingly, we collected several meteorological variables, including the amount of rainfall for the day (in cm), the atmospheric temperature (in °C), the humidity (in %), and the atmospheric pressure (in atmospheres); these public data were obtained from the France national meteorological office (Météo France).

Each quantitative variable was described by a density curve. This curve was compared with a normal distribution, with its mean and variance matching those observed in the study sample. We also plotted pollutant concentrations as a function of the day of the year; a generalized additive model regression curve was produced for visualization only. Associations between CC16, 8-OHdG, 4-HNE or FOPs and air pollutants were studied with linear regressions adjusted for age, body mass index, and the previous day’s rainfall, humidity, atmospheric temperature, and pressure. As all biomarkers had a distribution that was close to log-normal, each model’s outcomes were log-transformed. Multicollinearity was assessed for each model and variance inflation factors of less than 2 were considered acceptable. We performed a sensitivity analysis using robust linear regressions because some models’ residuals were not normally distributed with the presence of extreme values, which might have biased the coefficients. Specifically we used models that didn’t use the standard minimize the mean square error, but instead use an MM estimator (Khotimah et al., 2020), less sensitive to extreme values.

Regression coefficients were expressed as the percentage change for 10 μg/m3 change in pollutant concentration. Statistical significance was set at *p* ≤ 0.05.

The power to replicate other studies’ beta coefficients was obtained by calculating the Pearson correlation coefficient we would have observed in our study if our beta coefficient had been the same as theirs, and then using Cohen’s method (Cohen, 1977).

## Results

Out of a total of 3275 ELISABET participants, 101 withdrew their consent to the use of personal data. In total, 980 participants met our inclusion criteria. Two CC16 assays, one FOP assay, and 22 4-HNE assays failed to produce interpretable results. All the participants had data for the covariates.

After excluding participants with missing exposure data or with no valid biomarker assays, and 10 participants with biomarker samples outside the detection range, our analysis of short-term air pollution exposure included 978 participants for CC16, 959 for 8-OHdG, 958 for 4-HNE, and 979 for FOPs. Our analysis of residential air pollution exposure included 977 participants for CC16, 958 participants for 8-OHdG, 957 for 4-HNE, and 978 for FOPs.

The median age of the study population was 54, and the mean body mass index was 26 kg/m^2^ (Table 1).

**Table 1:** Population characteristics

	N = 980^a^
Age (years)	54.14 (47.71; 59.89)
Sex	
Males	450 (45.92%)
Females	530 (54.08%)
Body mass index (kg/m^2^)	26 (23.27; 29)
Socio-educational level	
5 or more years of higher education	262 (26.73%)
2 to 4 years of higher education	227 (23.16%)
No higher education	417 (42.55%)
No secondary education	74 (7.55%)
CC16 (pg/mL)	0.92 (0.64; 1.35)
8-OHdG (ng/mmol creatinine)	0.76 (0.58; 0.97)
4HNE (pg/mL)	0.96 (0.69; 1.33)
FOP 320 nm (FI/mL)	18.54 (15.75; 23)
FOP 360 nm (FI/mL)	85.33 (64.94; 104)
FOP 400 nm (FI/mL)	101.17 (84.54; 121)
Short-term PM_10_ (μg/m^3^)	22.50 (16.50; 36)
Short-term NO_2_ (μg/m^3^)	24.13 (18.23; 32)
Short-term O_3_ (μg/m^3^)	60.64 (43.17; 74)
Residential PM_10_ (μg/m^3^)	26.88 (25.48; 28)
Residential NO_2_ (μg/m^3^)	25.64 (22.19; 28)

^a^Median (IQR); n (%)

CC16, Club cell protein 16; 4HNE, 4-Hydroxynenonenal; 8-OHdG, 8-Hydroxyguanosine; FOP, Fluorescent oxidation products; FI/mL, Fluorescence intensity by milliliter

The median level of short-term PM_10_ exposure was 22.5 μg/m^3^, and the maximum level was 92.5 μg/m^3^ (Table 1). For NO_2_, the median level of short-term exposure was 24.1 μg/m^3^, and the maximum was 72.8 μg/m^3^. For O_3_, the median level of short-term exposure was 60.7 μg/m^3^, and the maximum was 178.3 μg/m^3^. The median levels of residential exposure were 26.9 μg/m^3^ for PM_10_ with values ranging from 23.0 to 43.0, and 25.6 μg/m^3^ for NO_2_ with values ranging from 15.5 to 80.4. The pollutant concentrations as a function of the day of the year are shown in Supplemental Fig. 1.

For short-term exposure, PM_10_ and NO_2_ were positively correlated (r = 0.692), while O_3_ was negatively correlated with NO_2_ (r = − 0.489) and PM_10_ (r = − 0.126). For residential exposure, the coefficient for the correlation between PM_10_ and NO_2_ was 0.773, all *p* < 0.001.

None of the associations between CC16 and daily (short-term) exposure to PM_10_, NO2 or O3 was significant (Table 2). Similarly, none of the associations between CC16 and the mean annual residential exposure to PM_10_ or NO_2_ was significant (Table 3).

**Table 2:** Associations between short-term air pollution exposure and plasma or urinary biomarker levels

Biomarker	PM_10_		NO_2_		O_3_	
	Percentage change for 10 µg/m3	*p*	Percentage change for 10 µg/m3	*p*	Percentage change for 10 µg/m3	*p*
FOP (320 nm)	2.49% [0.64%; 4.37%]	**0.008**	2.77% [− 0.15%; 5.78%]	0.063	− 0.84% [− 2.62%; 0.98%]	0.364
FOP (360 nm)	1.84% [− 0.21%; 3.93%]	0.079	0.58% [− 2.60%; 3.86%]	0.725	− 0.21% [− 2.21%; 1.82%]	0.837
FOP (400 nm)	− 0.10% [− 1.47%; 1.28%]	0.881	− 0.38% [− 2.52%; 1.80%]	0.728	− 0.25% [− 1.60%; 1.12%]	0.721
CC16	0.26% [− 2.34%; 2.92%]	0.849	− 1.39% [− 5.39%; 2.78%]	0.507	1.86% [− 0.75%; 4.55%]	0.164
Urinary 8− OHdG	− 0.59% [− 2.46%; 1.30%]	0.536	− 0.74% [− 3.71%; 2.32%]	0.632	0.38% [− 1.50%; 2.31%]	0.692
4− HNE	− 0.88% [− 3.40%; 1.71%]	0.501	− 1.08% [− 5.03%; 3.04%]	0.601	0.60% [− 1.95%; 3.21%]	0.647

Concerning FOPs, a significant association between daily (short-term) exposure to PM10 and FOPs excited at 320 nm was observed, with a 2.49% [0.64%; 4.37%] (p = 0.008) increase in FOP levels per 10 μg/m3 increment in PM10

Log-linear regressions adjusted for age, body mass index, and the previous day’s rainfall, humidity, atmospheric temperature, and pressure

CC16, Club cell protein 16; 4HNE, 4-Hydroxynenonenal; 8-OHdG, 8-Hydroxyguanosine; FOP, Fluorescent oxidation products

**Table 3:** Associations between residential air pollution exposure and plasma or urinary biomarker levels

Biomarker	PM_10_		NO_2_	
	Percentage change for 2 µg/m3	p	Percentage change for 5 µg/m3	p
FOP (320 nm)	0.74% [− 2.34%; 3.91%]	0.641	− 0.48% [− 3.07%; 2.18%]	0.719
FOP (360 nm)	2.13% [− 1.34%; 5.72%]	0.231	0.56% [− 2.35%; 3.55%]	0.710
FOP (400 nm)	0.90% [− 1.43%; 3.28%]	0.452	− 0.01% [− 1.97%; 2.00%]	0.994
CC16	− 1.61% [− 5.90%; 2.87%]	0.474	− 2.03% [− 5.67%; 1.75%]	0.287
Urinary 8-OHdG	0.35% [− 2.84%; 3.64%]	0.833	0.37% [− 2.35%; 3.17%]	0.790
4-HNE	1.12% [− 3.21%; 5.63%]	0.618	− 0.66% [− 4.28%; 3.09%]	0.724

Log-linear regressions adjusted for age, body mass index, and the previous day’s rainfall, humidity, atmospheric temperature, and pressure

CC16, Club cell protein 16; 4HNE, 4-Hydroxynenonenal; 8-OHdG, 8-Hydroxyguanosine; FOP, Fluorescent oxidation products

None of the associations between 4-HNE or 8-OHdG, and daily (short-term) exposure to PM_10_, NO_2_ or O3 was significant (Table 2). Similarly, none of the associations between 4-HNE or 8-OHdG, and mean annual residential exposure to PM_10_ or NO_2_ was significant (Table 3).

Concerning FOPs, a significant association between daily (short-term) exposure to PM_10_ and FOPs excited at 320 nm was observed, with a 2.49% [0.64%; 4.37%] (*p* = 0.008) increase in FOP levels per 10 µg/m^3^ increment in PM_10_ (Table 2).

No other associations between FOPs and either short-term (Table 2) or residential exposure (Table 3) to the studied pollutants were found.

None of the associations between CC16, 4-HNE, 8-OHdG or FOPs and daily (short-term) exposure to PM_10_, NO_2_ or O3 was significant in the robust sensitivity analysis (Supplemental Table 1); this was notably true for the association between daily exposure to PM_10_ and FOPs (320 nm) mentioned in the previous section. Similarly, none of the robust models featured associations between CC16, 4-HNE, 8-OHdG or FOPs and mean annual residential exposure to PM_10_ or NO_2_ (Supplemental Table 2).

## Discussion

In this study of a large, population-based sample of middle-age adults, whilst naive models found a single association between short term PM_10_ exposure and FOP (320 nm) levels (+ 2.49% per 10 µg/m^3^ [0.64%; 4.37%], *p* = 0.008), this was explained by the model’s invalidity and wasn’t confirmed by the robust models. We did not observe any other significant associations between CC16, 8-OHdG, 4-HNE or FOPs and residential or short-term exposure to the studied air pollutants.

Several previous studies found associations between CC16 levels and pollutant exposure. One study found a negative association between NO_2_ exposure (mean(sd) 20.7 (11.3) µg/m^3^) at birth and the change in CC16 level in the longitudinal analysis (Beamer et al., 2019), with an observed 4.8% (0.7 to 8.6) decrease between the ages of 6 and 32; however, the effect was principally concentrated in black participants (n = 26, with a decrease of 29.6%, (13.2 to 42.9)). Another study found a positive association between short-term PM_10_ exposure (mean(sd) 30.6 (17.0) µg/m^3^) and the CC16 level in a sample of 825 adolescents (Provost et al., 2014), with a 0.21 (0.10 to 0.32) µg/L increase in CC16 per 5 µg/m^3^ increment in PM_10_. Our study had a power of 99.97% for detecting this effect. In our adult population, we did not replicate the results reported by others for children and adolescents. It might be that the effect of air pollution on CC16 is weaker in less vulnerable populations. Our study is consistent with two previous studies with smaller sample sizes. A Dutch study (Zuurbier et al., 2011) on 34 subjects found no association between PM_10_ (mean, 28 μg/m^3^) and CC16. Similarly an American study (Zhang et al., 2022) on 218 subjects found no association between neither NO_2_ nor PM_2.5_ and CC16.

Concerning 8-OHdG, a Taiwanese study (Lu et al., 2007) made repeated measurements in a sample of 76 young students and found a positive association between short-term outdoor O_3_ levels (range 14.5 to 96.6 µg/m^3^) and the urinary 8-OHdG level, with a 2.2% (0.9 to 3.5) increase in 8-OHdG per 8.9 µg/m^3^ increment in O_3_. Again, our present study of adults did not replicate this finding, even though it had a power of 95% for detecting this effect size. However, our study is consistent with a Chinese study(Mu et al., 2021) of 4697 adults that did not find an association between long-term PM_2.5_ exposure and 8-OHdG, with a 2.00% increase of 8-OHdG levels (− 0.40 to 4.46) per 10 µg/m^3^. One explanation for this disparity would be a weaker effect of air pollution on 8-OHdG levels in adults than in younger subjects.

There are no human studies on the impact of air pollution on 4-HNE levels. However, two mouse studies (Cacciottolo et al., 2020; Cheng et al., 2016) reported significant increases in 4-HNE (+ 25%, *p* < 0.01 and + 100%, *p* < 0.001) following exposure to extremely high levels of nano-particulate matter (343 and 300 μg/m^3^). Our study did not replicate these findings, likely due to physiological differences between species or the much lower pollution levels in our population. There is some evidence in the literature of an association between long-term air pollution exposure and FOP levels. A case–control study (Havet et al., 2019) of adults with asthma found a 0.04 (0.001 to 0.08) fluorescence intensity increment in FOP levels per 10 μg/m^3^ of mean annual exposure to PM_10_. Even though our population-based study had a 99.7% power for detecting this effect size, we did not observe this association. This disparity might be explained by a weaker effect of air pollution on FOPs in healthy adults than in adults with asthma. Our results are consistent with the absence of an association (+ 0.53 FI/mL (− 0.29 to + 1.35) per μg/m^3^ of PM_2.5_) between short-term PM_2.5_ exposure and FOP levels in a study of 236 Caucasian, non-smoking, male trucking industry workers (Hart et al., 2012). The present study had several strengths. Firstly, we studied a large, population-based sample. Secondly, our biomarker assays were subject to robust quality control procedures. Thirdly, we evaluated both short-term exposure and 1 to 3 years average residential exposure, which are suspected to produce different health effects (Beverland et al., 2012).

Our study also had several limitations, some of which were related to the design of the ELISABET cohort study and have been described previously (Darras-Hostens et al., 2022; Dauchet et al., 2018; Quach et al., 2015; Riant et al., 2018). Firstly, this was cross-sectional study with no repeated measurements; the latter would have enabled us to control more effectively for inter-individual variability—especially in the analysis of short-term exposure. This lack of repeated measurements probably reduced the study’s power and so might have masked the pollutants’ true effects on the selected biomarkers. Another limitation was the exclusion of patients outside the assays’ detection ranges, although the very small number (10) of subjects outside the limits of detection may have limited the impact on the result.

Lastly a key limitation to this study was the unavailability of data on PM_2.5_ and the composition of the PM. Indeed, the WHO has identified PM_2.5_ as a more reliable indicator of air quality compared to PM_10_ (“Health Risks of Air Pollution in Europe—HRAPIE Project. Recommendations for Concentration–Response Functions for Cost–Benefit Analysis of Particulate Matter, Ozone and Nitrogen Dioxide” 2013; “WHO Guidelines for Indoor Air Quality: Selected Pollutants,” n.d.). However, some research suggests that PM_10_ may have stronger associations with insulin resistance than PM_2.5_ (Wolf et al., 2016). Investigating both PM10 and PM2.5 would provide valuable insights. Nonetheless, these two pollutants often show a degree of correlation. For instance, in Augsburg, Germany, the correlation between measured PM_2.5_ and PM_10_ levels was found to be 0.74 (Wolf et al., 2017), suggesting that PM_10_ levels could serve as an indirect measure of PM_2.5_ exposure.

Our data did not provide evidence of any significant associations between air pollution and the selected biomarkers. Nevertheless, current scientific and medical knowledge suggests that oxidative stress is involved in pathophysiological mechanisms leading to lung and bronchial damage (Mu et al., 2021). Our study had adequate statistical power to detect associations previously observed in vulnerable populations. However, the low variability of long-term exposure and the absence of repeated measurements might have limited our ability to detect small effects. Our results need to be confirmed in studies with repeated measures and/or a greater range of exposure levels.

## Conclusion

Our results for a large sample of adults from the general population in the Lille urban area (France) did not provide evidence of an association between short-term or residential exposure to PM10, NO2 and ozone and CC16, 8-OHdG, 4-HNE and FOP biomarker levels. Even though our study had sufficient statistical power, we did not replicate previous reports of associations between air pollution and these biomarkers in potentially more vulnerable populations (e.g. children, teenagers, and people with asthma). These findings suggest that the general adult population may be less sensitive to air pollutant exposure.

## Supplementary Information

Below is the link to the electronic supplementary material.

Supplementary file1 (SVG 26 KB)

Supplementary file2 (DOCX 28 KB)

Supplementary file3 (DOCX 28 KB)

## Data Availability

No datasets were generated or analysed during the current study.
