# Supplementary material for: Associations between air pollution and biomarkers of oxidative stress and lung damage in a large population-based sample of non-smoking adults in northern France
Source: Environ Geochem Health. 2025 Apr 12;47(5):166. doi: 10.1007/s10653-025-02472-2 (PMC11993482; doi:10.1007/s10653-025-02472-2)
Supplement: Supplementary file 2 — Supplementary file2 (DOCX 28 KB) [file 10653_2025_2472_MOESM2_ESM.docx]

|  | PM_10_ | | NO_2_ | | O_3_ | |
| --- | --- | --- | --- | --- | --- | --- |
| Biomarker | Percentage change for 10 µg/m³ | p | Percentage change for 10 µg/m³ | p | Percentage change for 10 µg/m³ | p |
| FOP (320 nm) | 0.38% [-0.93%; 1.70%] | 0.574 | -0.33% [-2.50%; 1.89%] | 0.768 | 0.03% [-1.29%; 1.36%] | 0.967 |
| FOP (360 nm) | 0.73% [-1.00%; 2.49%] | 0.409 | -0.75% [-3.92%; 2.51%] | 0.647 | -0.02% [-1.88%; 1.88%] | 0.982 |
| FOP (400 nm) | -0.10% [-1.33%; 1.15%] | 0.878 | -0.60% [-2.77%; 1.61%] | 0.590 | -0.81% [-2.18%; 0.58%] | 0.253 |
| CC16 | 0.57% [-2.07%; 3.28%] | 0.677 | -1.33% [-5.42%; 2.93%] | 0.534 | 2.01% [-0.80%; 4.89%] | 0.163 |
| Urinary 8-OHdG | -0.48% [-2.32%; 1.40%] | 0.616 | -0.10% [-3.17%; 3.06%] | 0.948 | 0.54% [-1.28%; 2.39%] | 0.562 |
| 4HNE | -0.40% [-3.03%; 2.29%] | 0.766 | -1.89% [-5.99%; 2.39%] | 0.382 | 1.21% [-1.54%; 4.04%] | 0.391 |

Supplementary Table 1. Associations between short-term air pollution exposure and plasma or urinary biomarker levels

Log-linear robust regressions adjusted for age, body mass index, and the previous day’s rainfall, humidity, atmospheric temperature, and pressure.

Abbreviations

CC16 : Club cell protein 16

4HNE : 4-Hydroxynenonenal

8-OHdG : 8-Hydroxyguanosine

FOP : Fluorescent oxidation products
