# Supplementary material for: Associations between air pollution and biomarkers of oxidative stress and lung damage in a large population-based sample of non-smoking adults in northern France
Source: Environ Geochem Health. 2025 Apr 12;47(5):166. doi: 10.1007/s10653-025-02472-2 (PMC11993482; doi:10.1007/s10653-025-02472-2)
Supplement: Supplementary file 3 — Supplementary file3 (DOCX 28 KB) [file 10653_2025_2472_MOESM3_ESM.docx]

|  | PM_10_ | | NO_2_ | |
| --- | --- | --- | --- | --- |
| Biomarker | Percentage change for 2 µg/m³ | p | Percentage change for 5 µg/m³ | p |
| FOP (320 nm) | 1.42% [-0.97%; 3.86%] | 0.246 | -0.01% [-2.27%; 2.29%] | 0.990 |
| FOP (360 nm) | 0.08% [-3.58%; 3.87%] | 0.967 | -0.79% [-4.26%; 2.80%] | 0.662 |
| FOP (400 nm) | 0.24% [-2.01%; 2.53%] | 0.839 | -0.49% [-2.43%; 1.50%] | 0.629 |
| CC16 | -0.87% [-4.90%; 3.33%] | 0.681 | -1.90% [-4.98%; 1.28%] | 0.239 |
| Urinary 8-OHdG | 0.36% [-3.00%; 3.82%] | 0.838 | 0.69% [-2.11%; 3.58%] | 0.632 |
| 4HNE | 0.80% [-3.56%; 5.37%] | 0.723 | -1.14% [-4.46%; 2.31%] | 0.513 |

Supplementary Table 2. Associations between residential air pollution exposure and plasma or urinary biomarker levels

Log-linear robust regressions adjusted for age, body mass index, and the previous day’s rainfall, humidity, atmospheric temperature, and pressure.

Abbreviations

CC16 : Club cell protein 16

4HNE : 4-Hydroxynenonenal

8-OHdG : 8-Hydroxyguanosine

FOP : Fluorescent oxidation products
